# Triage and vital signs in a population discharged from and readmitted to the ED

**DOI:** 10.1186/1757-7241-21-S2-A10

**Published:** 2013-09-09

**Authors:** Ida Helsø, Helle Ipsen, Claus Heinecke, Hanne Jørsboe

**Affiliations:** 1 Medical Department, Nykobing F Hospital, Denmark; 2 Emergency Department, Nykobing F Hospital, Denmark; 3 Department of Quality Improvement, Nykobing F Hospital, Denmark

## Background

In our community hospital about 20% of the patients are readmitted within 30 days to the emergency room, where clinical effectiveness and patient safety depend on the triage process including observation of vital signs. Therefore, the study was performed to describe this population and to compare the triage score and vital signs at the first contact and at readmission.

## Methods

Patients were identified from a national database and evaluated through an audit of electronic patient files with registration of the following criteria; triage-level, vital signs, medical problems and diagnosis, supplemented with the vital signs monitored the last day before discharge from the first hospitalization. The vital signs were summarized to a standardized score called BOS. The triage system is a 5 point-scale in colours, where 1 compared to “red” resuscitation. Data were evaluated with a Mann-Whitney non-parametric statistic for paired data.

## Results

A sample of 50 cases were included (26 F, 24 M), mean age 57 years (21-92) of which 64% of the patients had co-morbidity. Most of the patients were admitted with symptoms of abdominal pain (20%), dyspnoea (14%) and alcohol related disease (10%). At readmission, 58% patients had related symptoms. The average triage score at the primarily contact were urgent (mean: 3 (1-4)) and BOS score 1 (0-5), which was reduced through stabilization to 0 (0-3) (p<0.05). At readmission, the triage score was 3 (2-4) and BOS level was increased to 1 (0-5). 22% of the patients evaluated by triage had a higher degree of acuity compared to the first contact, supported by 33% of the patients were evaluated worsened by vital signs (BOS).

## Conclusion

These data suggest a relative young population is readmitted to the ED compared to international studies. A part of these patients with a high degree of co- morbidity, were evaluated urgent by triage and BOS at readmission, despite stabilization before discharge from the first hospitalization. Further audit will be extended to a larger population.

